# High Prevalence of Post-Traumatic Stress Symptoms in Relation to Social Factors in Affected Population One Year after the Fukushima Nuclear Disaster

**DOI:** 10.1371/journal.pone.0151807

**Published:** 2016-03-22

**Authors:** Takuya Tsujiuchi, Maya Yamaguchi, Kazutaka Masuda, Marisa Tsuchida, Tadashi Inomata, Hiroaki Kumano, Yasushi Kikuchi, Eugene F. Augusterfer, Richard F. Mollica

**Affiliations:** 1 Faculty of Human Sciences, Waseda University, Tokorozawa-shi, Saitama, Japan; 2 Waseda Institute of Medical Anthropology on Disaster Reconstruction, Shinjuku, Tokyo, Japan; 3 Harvard Program in Refugee Trauma, Harvard Medical School, Cambridge, Massachusetts, United States of America; 4 Department of Social Welfare, The International University of Kagoshima, Kagoshima, Kagoshima, Japan; 5 Idente Foundation for Studies and Research, Rome, Italy; 6 Shinsai Shien Network Saitama, Saitama Sogo Law Firm, Saitama-shi, Saitama, Japan; 7 Graduate School of Asia-Pacific Studies, Waseda University, Shinjuku-ku, Tokyo, Japan; University of Geneva, SWITZERLAND

## Abstract

**Objective:**

This study investigated post-traumatic stress symptoms in relation to the population affected by the Fukushima Nuclear Disaster, one year after the disaster. Additionally, we investigated social factors, such as forced displacement, which we hypothesize contributed to the high prevalence of post-traumatic stress. Finally, we report of written narratives that were collected from the impacted population.

**Design and Settings:**

Using the Impact of Event Scale-Revised (IES-R), questionnaires were sent to 2,011 households of those displaced from Fukushima prefecture living temporarily in Saitama prefecture. Of the 490 replies; 350 met the criteria for inclusion in the study. Multiple logistic regression analysis was performed to examine several characteristics and variables of social factors as predictors of probable post-traumatic stress disorder, PTSD.

**Results:**

The mean score of IES-R was 36.15±21.55, with 59.4% having scores of 30 or higher, thus indicating a probable PTSD. No significant differences in percentages of high-risk subjects were found among sex, age, evacuation area, housing damages, tsunami affected, family split-up, and acquaintance support. By the result of multiple logistic regression analysis, the significant predictors of probable PTSD were chronic physical diseases (OR = 1.97), chronic mental diseases (OR = 6.25), worries about livelihood (OR = 2.27), lost jobs (OR = 1.71), lost social ties (OR = 2.27), and concerns about compensation (OR = 3.74).

**Conclusion:**

Although there are limitations in assuming a diagnosis of PTSD based on self-report IES-R, our findings indicate that there was a high-risk of PTSD strongly related to the nuclear disaster and its consequent evacuation and displacement. Therefore, recovery efforts must focus not only on medical and psychological treatment alone, but also on social and economic issues related to the displacement, as well.

## Introduction

The Great East Japan Earthquake, GEJE, occurred on March 11^th^, 2011. The magnitude 9.0 earthquake and subsequent massive tsunami struck the Pacific coast of northeastern Japan, leading to the Fukushima nuclear disaster, including the meltdown of reactor № 1 of Fukushima Daiichi Nuclear Power Plant on March 12^th^, and three additional reactors on March14^th^ and 15^th^.

As a result of the GEJE, over 15,800 people died, over 3,200 people went missing, over 6,000 people were injured, and more than 321,000 people were forced to evacuate, thus the GEJE is one of the largest disasters ever in Japan [1]. The GEJE has drawn comparisons to the Chernobyl nuclear disaster in 1986, due to both disasters being major nuclear disasters, including radiological contamination to the surrounding environment.

The government of Japan declared a nuclear emergency on March 11^th^, forcing the evacuation of citizens in Fukushima Prefecture within a 20km radio of the disaster zone on March 12^th^, and those living between 20 and 30km were urged to evacuate on March 25^th^. Finally, on April 22^th^, the high risk level of contamination required the expansion of the evacuation zone to 30km zone of the disaster site. Out of 150,000 evacuees from Fukushima Prefecture, 90,000 were relocated to another region within the Fukushima Prefecture, and about 60,000 residents were relocated to other prefectures, such as Yamagata, Tokyo, Niigata and Saitama.

A review of the natural and technological disaster literature reveals that post-traumatic stress disorder (PTSD) levels range from 30–40% in directly exposed victims and 5–10% in general population [2]. One year after the GEJE, the Cabinet Office of Policy for Suicide Prevention published pamphlets to promote a better understanding of mental healthcare for disaster affected citizens [3]. These pamphlets emphasized, the importance of psychological support to particularly vulnerable groups, including women, children, elderly, handicapped, those with pre-existing mental disorders patients, and the impoverished [4].

After the GEJE, there were several reports of medical support team actions including mental health care [5, 6]. Among the studies of psychological distress in rescue workers, Fushimi [7] reported 1.7% of firefighters met the criteria for high risk of PTSD, Nishi et al. [8] reported on the members of the Disaster Medical Assistance Team (DMAT), that the number of hours of watching earthquake television news was predictive of PTSD symptoms, with a mean score of 6.8 on the Impact of Event Scale-Revised (IES-R) of the participants. Matsuoka et al. [9] also studied the DMAT, and found concerns about radiation exposure associated with psychological distress. Using the IES-R, they found that in the concerned group, the score was 22.3, and in the not concerned group, the score was 8.5. Shigemura et al. [10, 11] reported the psychological distress in Fukushima nuclear power plant workers two to three months after the disaster assessed by IES-R. The study showed higher rates of post-traumatic stress responses in the Daiichi power plant workers (30% broadly defined PTSD) versus the Daini power plant workers (19%) (it is important to note that the explosion of the Daiichi power plant may have attributed for the difference in PTSD scores).

While several previous studies systematically examined the magnitude of the impact on mental health after the GEJE [12–14], there is only one study which examined the impact of the GEJE and nuclear disaster on the affected population. Kukihara et al., [15] investigated the evacuated residents housed in temporary housing units from Hirono-city, Fukushima prefecture, nine months after the disaster, the mean score of the IES-R was 26.7, and 53.5% exhibited clinically concerning symptoms of PTSD, and 33.2% indicated clinical PTSD symptoms. Thus, the study of the affected population suffering the long term impact of the disaster have higher rates of PTSD than rescue workers or power plant workers. Since, Nomura [16] reported the socio-economic factors including the loose of livelihood and delayed evacuation besides the direct damage due to tsunami disaster related to higher prevalence of PTSD, it is important to focus and expand the scope of disaster research on the causes and factors explaining this higher rate of PTSD.

Given that affected population showed high prevalence of probable PTSD, we hypothesized that several socio-economic factors as contributors to the high prevalence of PTSD one year after the nuclear disaster. We used the IES-R to investigate the values of PTSD symptoms one year after the disaster on all the evacuees from the Fukushima nuclear disaster living in Saitama prefecture.

## Method

### Data Collection and Sample

This was a joint survey conducted by Waseda University and a private disaster support group “Shinsai Shien Network (SSN)” in Saitama prefecture. A questionnaire was designed and sent out to all 2011 households of Fukushima evacuees living at Saitama prefecture, through the designated local government advertisement flyer, supported by Fukushima Prefecture Disaster Countermeasures Headquarters Out-side Prefecture Refugee Support Team. The data were collected by mailing between March and April, 2012. The sample in this survey was all the households which were reportedly evacuated from the inhabitable Fukushima`s nuclear disaster area to government settled temporarily living in Saitama prefecture. One representative member per household replied to our questionnaires. Questionnaires with incomplete data were excluded.

### Study Instrument

Socio demographic data: The following socio-demographic data was gathered: gender; age, less than and more and equal 60 years old; evacuation area such as hazard area, evacuation prepared area, deliberate evacuation area, and other area of Fukushima prefecture; housing damages, such as completely damaged, half damaged, partially damaged, and no damaged; if there were any kind of tsunami affection classified as affected, partially affected, and no affection.

Health status: We asked for chronic physical and mental diseases already present before the disaster and still ongoing. Participants were asked to complete the Japanese-language version of the Impact of Event Scale-Revised (IES-R-J). The reliability and validity of IES-R-J has been confirmed by Asukai et al. [17] in four different trauma populations: workers with mixed lifetime events, survivors of an arsenic poisoning, the Great Hanshin-Awaji earthquake, and Tokyo Metro sarin attack. In these studies, psychiatrists and psychologists evaluated PTSD diagnoses of subjects with the PTSD module of the Structured Clinical Interview for DSM and Clinician Administered PTSD Scale (CAPS), and determined IES-R-J as a useful instrument to evaluate survivors with PTSD symptoms as a clinical concern after various kinds of traumatic events [17]. IES-R includes 22-items self-report scale assessing the frequency of the symptoms of PTSD; intrusive, avoidance and hyper arousal symptoms following trauma. IES-R is the most internationally used measure in the disaster field, and psychometric validation studies were shown in different cultural contexts [18]. Creamer M, et al. [19] used a cut-off score of ≥33 to identify probable PTSD, which is the cut-off recommended to provide the best diagnostic accuracy to identify persons with high levels of posttraumatic stress. However, the authors recognize that the IES-R does not render a diagnosis of PTSD, but indicates probable PTSD and not a clinical diagnosis of PTSD. In IES-R-J, the cut-off point of narrowly defined PTSD is 29/30, and broadly defined PTSD is 24/25 [17]. Even though this cut-off of 30 slightly less conservative than Creamer’s cut-off score of 33 [19], the narrowly defined PTSD-positive group is statistically defined as including full PTSD alone while broadly defined PTSD included full and partial PTSD together [17].

Socio-economic disaster related consequences: We asked for the presence or absence of the following factors: worries for livelihood; lose of jobs; family split-up due to displacement after the disaster because of jobs, education needs, or space restriction; lose of social ties; lose of acquaintance support; useful information for daily refugee life; and concerns for nuclear disaster compensation.

Narratives: In addition we collected written narratives from participants so that they could express in their own words the impact of the disaster and subsequent displacement. While this report was not quantitatively rated, we consider this data as valuable as it provides insights into the suffering as it may explain the high prevalence of PTSD.

### Statistical Analysis

We calculated the mean±SD score of the IES-R, and determine the percentage of high level PTSD symptoms by 29/30 cutoff point [17] of IES-R for several characteristics and variables of social factors. Chi-square test was used to compare the percentage of probably PTSD for each socio- demographic variables, health status and socio-economic disaster related consequences.

Multiple logistic regression analysis was performed to examine individual predictors for PTSD. Covariates were considered in the multivariate regression model by stepwise variable selection in which bivariate chi-square p-values were less than 0.1. Differences in log likelihood (p<0.05) were used to determine whether variables would be retained in subsequent models. Each odds ratio was shown with the key predictor variables in the final models.

All the statistical significance was set at p<0.05 and SPSS version 20 software (IBM Inc., Tokyo, Japan) was used to analyze data.

### Ethical considerations

The participants had their rights protected in the spirit of the Declaration of Helsinki. The research project obtained the approval (No. 2012–011) of the Ethics Committee of Waseda University. The nature of the research, assurances of confidentiality, and contact numbers for counseling are explained in a covering letter. Participants returned the questionnaires anonymously in a pre-paid envelope.

## Results

### Sample Information

490 questionnaires were collected by mailing with an overall cooperation rate for the survey of 24.4 percent. However, 140 questionnaires had to be excluded from statistical analysis because of failure to complete the questionnaire, leaving a final sample of 350 households available for data analysis. The final sample of 350 households was composed of 163 males and 187 females. The mean (±SD) age was 54.2±15.2 years. The number of subjects under the age of 60 years old was 215, and 60 or over was 135. The number of evacuees from Hazard Area was 299, from Evacuate Prepared Area in case of emergency was 38, from Deliberate Evacuation Area was 8, and from other area was 3. As for housing damage, 32 households were completely destroyed, 48 were half destroyed, 181 were partially damaged, and 53 were not damaged. Regarding those affected by the tsunami, 24 were affected, 6 were partially affected, and 304 were not affected.

### Prevalence of probable PTSD

The mean score of the Impact of Event Scale-Revised (IES-R) was 36.15±21.55, with 59.4% of the evacuees indicate the prevalence of probable PTSD. The sub-scale of mean IES-R was 13.94±8.38 for intrusion, 11.86±7.97 for avoidance, and 10.35±6.59 for hyper-arousal.

### Bivariate Analyses

No significant differences were found by chi-square test among gender, age, evacuation area, housing damages, damage by tsunami, family split-up, and acquaintance support (Table 1). The significant high percentage of PTSD were demonstrated in the groups with chronic physical diseases (p = .045), chronic mental diseases (p = .008), worries about livelihood (p < .001), lost jobs (p = .042), lost social ties (p = .010), absence of getting useful information (p = .026), and concerns about compensation (p < .001).

**Table 1:** Bivariate associations between characteristics of the respondents and probable-PTSD.

			Probable-PTSD		
Characteristics		N^a^	% ^b^	Χ2^c^	*p*-value
Total		350	59.4		
Gender				2.25	0.134
	Male	163	55.2		
	Female	187	63.1		
Age				0.71	0.399
	<60 years old	215	57.7		
	> = 60 years old	135	62.2		
Evacuation area				2.00	0.573
	Hazard area	299	60.2		
	Evacuation prepared area	38	55.3		
	Deliberate evacuation area	8	37.5		
	Other	3	66.7		
Housing damages				3.55	0.315
	Completely damaged	32	59.4		
	Half damaged	48	66.7		
	Partially damaged	181	60.8		
	No damaged	53	49.1		
Tsunami affection				0.19	0.908
	Affected	24	58.3		
	Partially affected	6	50.0		
	No affection	304	58.9		
Chronic physical diseases				4.00	0.045*
	Yes	173	64.7		
	No	177	54.2		
Chronic mental diseases				7.14	0.008**
	Yes	29	82.8		
	No	321	57.3		
Worries livelihood				13.4	<0.001***
	Yes	243	65.4		
	No	98	43.9		
Lost jobs				4.12	0.042*
	Yes	171	64.9		
	No	170	54.1		
Family split-up				0.06	0.807
	Yes	195	60.0		
	No	155	58.7		
Lost social ties				6.67	0.010*
	Yes	41	78.0		
	No	309	57.0		
Acquaintance support				0.72	0.396
	Yes	220	57.7		
	No	125	62.4		
Useful information				4.93	0.026*
	Yes	203	53.7		
	No	140	65.7		
Concerns compensation				12.25	<0.001***
	Yes	307	62.9		
	No	43	34.9		

^a^ Numbers of each groups. Numbers may not add up to 350 because not all the respondents answered all the questions.

^b^ Percentage of the numbers of probable PTSD scored over 29/30 cutoff point of IES-R.

^c^ The chi-square test was used for comparison; p-values are two-tailed.

*p<0.05.

**p<0.01.

***p<0.001.

### Multivariate Analyses

The results of multiple logistic regression analysis are shown in Table 2. Significant predictors of PTSD were chronic physical diseases (OR = 1.97, 95%CI = 1.21–3.23), chronic mental diseases (OR = 6.25, 95%CI = 1.96–19.95), worries about livelihood (OR = 2.27, 95%CI = 1.32–3.88), lost jobs (OR = 1.71, 95%CI = 1.05–2.78), lost social ties (OR = 2.27, 95%CI = 1.00–5.16), and concerns about compensation (OR = 3.74, 95%CI = 1.65–8.51).

**Table 2:** Multivariate association between characteristics of the respondents and probable-PTSD.

		Probable-PTSD		
Independent variables		Odds Ratio	95% CI	*p*-value
Chronic physical diseases				0.007**
	Yes	1.97	1.21–3.23	
	No	1.00		
Chronic mental diseases				0.002**
	Yes	6.25	1.96–19.95	
	No	1.00		
Worries livelihood				0.003**
	Yes	2.27	1.32–3.88	
	No	1.00		
Lost jobs				0.031*
	Yes	1.71	1.05–2.78	
	No	1.00		
Lost social ties				0.050
	Yes	2.27	1.00–5.16	
	No	1.00		
Concerns compensation				0.002**
	Yes	3.74	1.65–8.51	
	No	1.00		

*p<0.05.

**p<0.01.

## Discussion

### Prevalence of PTSD symptoms

This study demonstrates the increased prevalence rate of probable PTSD in residents evacuated from Fukushima prefecture one year after the nuclear disaster. The subjects reported PTSD symptoms based on the IES-R, the mean score using the IES-R was 36.15 points, indicating 59.4% had scores of 30 or higher.

There are many published articles that compare the prevalence of PTSD, but not comparable as they include different quality and measures. Therefore, we chose to compare all the previous studies with the same screening instrument IES-R in order to elaborate the explanation on the degree of severity of PTSD symptoms. All the following disaster researches in our discussion are shown in Table 3.

**Table 3:** Comparison of the impact of natural and human-made technological disasters measured by IES-R.

Study	Date of Disaster	Event	Type of Disaster	Number of Died people	Sample Type	Sample Size	Response Rate	Time After the Event ^a^	Score of Mean IES-R		Probable PTSD (%)
Kato et al. (2000) & Asukai et al. (2002) ^b^	1995	Great Hanshin-Awaji Earthquake, Japan	Natural	6434	Residents in Temporary housing or public permanent housing	86	Convenience sample	3.8 y	22.5±16.8; total		39.5
									41.4±13.6; PTSD		
									29.5±12.6; partial PTSD		
									18.9±16.0; non-PTSD		
Naoi et al. (2009) ^b^	2004	Nigata Chuetsu Earthquake, Japan	Natural	68	Residents	4,362	86%	3 m	14.7±14.8		21.0
						4,352	85%	13 m	14.3±13.8		20.8
Fushimi (2012) ^b^	2011	Great East Japan Earthquake	Natural	15,882	Firefighters in Akita City who had participated the rescue efforts at Tsunami area	117	99%	0 w	5.22±6.75		1.7
								2 w	2.06±3.48		0
								1 m	0.96±2.07		0
Matsuoka et al. (2012) ^b^	2011	Great East Japan Earthquake& Nuclear Disaster	Complex	15,882	Rescue workers of Disaster Medial Assistance Teams (n = 1816)	424	24%	1 m	Concern over radiation exposure (n = 39)	22.3±19.3; male	
										19.1±14.1; female	
									Not concern over radiation exposure (n = 385)	8.5±9.7; male	
										11.6±11.8; female	
Nishi et al. (2012) ^b^	2011	Great East Japan Earthquake & Nuclear Disaster	complex	15,882	Rescue workers of Disaster Medial Assistance Teams (n = 1816)	254	14%	4 m	6.8±8.4		
Shigemura et al. (2012) ^b^	2011	Great East Japan Earthquake & Nuclear Disaster	complex	15,882	Fukushima nuclear power plants workers (n = 1760)	1495	84.9%	2 to 3 m	Daiich workers (n = 885)		30
									Daini workers (n = 610)		19
Kukihara et al. (2014) ^b^	2011	Great East Japan Earthquake & Nuclear Disaster	complex	15,882	Residents of Hirono in temporary housing units (n = 458)	241	53%	9 m	26.7±17.9; Total		
									Clinical concern PTSD (n = 129)		53.5
									Clinical PTSD (n = 80)		33.2
Cetin et al. (2005) ^c^	1999	Marmara, Turkey Earthquake	Natural	17,127	Volunteer Rescue workers	434	70%	3 m	22.7±19.8		
Johannesson et al. (2011) ^c^	2004	Southeast Asian Earthquake Tsunami	Natural	227,000	Swedish Tourists (n = 10,501)	3,457	33%	14 m	25.65±18.80; high exposure		
									16.62±14.93; medium exp.		
									8.70±10.40; low exp.		
									36.48±18.51; family loss		
								3 y	18.13±16.24; high exp.		
									11.15±12.31; medium exp.		
									4.87± 7.34; low exp.		
									17.28±16.78; family loss		
Zhang et al. (2011) ^c^	2008	Wenchen Earthquake, China	Natural	69,227	Service recipients of the Institute of Psychology, living in temporary accommodation facilities	956	Convenience sample	1 m	43.39±10.86		82.6
Wang et al. (2011) ^c^	2008	Wenchen Earthquake, China	Natural	69,227	Collected by psychological relief workstation and a local general hospital	3,622	Convenience sample	3 m	26.7±18.0		31.4
Arnberg et al. (2011) ^d^	1994	MS Estonia (car-ferry) Disaster	Complex	852	Swedish domicile of passengers, crewmembers (n = 51)	39		3 m	42±21		44
						31		1 y	34±21		35
						24		3 y	34±27		38
						33	69%	14 y	33±27		27

^a^ Time after the disaster event when the study performed. w: week, m: month, y: year.

^b^ Natural and complex disasters in Japan.

^c^ Natural disasters in the world.

^d^ Human-made or technological disasters in the world.

Compared to other natural disasters in Japan, our data shows severe psychological distress. The Great Hanshin-Awaji Earthquake, magnitude 7.3 hit Kobe central city, in 1995 and caused 6,434 deaths and the evacuation of more than 300,000 people. In the study of temporary housing residents four years after the earthquake, the mean score of IES-R was 22.5, with 39.5% over broadly defined for probable PTSD. Finally 9.3% were diagnosed with PTSD by a Clinician-Administered PTSD Scale [17, 20] (Table 3). The Niigata Prefecture Chuetsu Earthquake of 2004 was the third largest disaster to hit Japan, with a magnitude of 6.8, killing 68 people and causing the evacuation of more than 100,000 people. Naoi [21] performed the epidemiological study 3 and 13 months after the Niigata earthquake and found that the mean score of 14 to 15 using the IES-R, which indicated about 21% of those examined had probable PTSD (Table 3).

We reviewed other earthquake and tsunami studies which reported high mean scores of IES-R in the world [21–25] (Table 3). Cetin M. et al. [22] reported 27.7 mean IES-R score of rescue workers in the Turkey earthquake of 1999 (Table 3). In a study by Zhang Y. et al. [23] of the Wenchen earthquake of 2008 in China, they found the most common traumatic experiences encountered were losing one's home (i.e., house had collapsed or was severely damaged; 37.2%, n = 356) and being trapped under the ruins of collapsed buildings (34.7%, n = 332). Bereavement was the third most common trauma encountered, with 29.7% (n = 284) of our participants reporting that one or more of their family members had been killed by the earthquake [23]. Regarding the prevalence of PTSD, the mean total score of IES-R was 43.39 and 790 participants (82.6%) had an IES-R score higher than 33, and could be identified as “probable PTSD” [23] (Table 3). Wang L. et al. [24] also evaluated the prevalence of possible PTSD of the Wenchen earthquake. Samples (n = 3622) were collected by psychological relief workstation and local general hospital, the mean score of IES-R was 26.7 and 31.4% had higher than 33 [24] (Table 3).

A study of the 2004 Southeast Asia Tsunami by Maegele et al. [25], reported that the World Health Organization estimated that up to 50% of the five million people affected by the tsunami would experience moderate to severe psychological distress and approximately 5% to 10% would develop more persistent problems, such as, depression, anxiety and PTSD [25]. In a study of Swedish tourist visiting Sri Lanka during the 2004 tsunami, Johannesson et al. [26], found that exposure was associated with increased levels of post-traumatic stress reactions even 3 years after the disaster. Those who were highly-exposed had approximately 3 times the IES-R scores of the low-exposed, and medium-exposed had 2 times the IES-R scores of the low-exposed. This indicates a dose-response relationship, which is in agreement with other studies [26] (Table 3).

In accordance with the theory of stressor dose-response relationship [27] in the incident rate of PTSD, the mean score of IES-R reflects the seriousness of the disaster and the exposure to the disaster. However, in our survey of 350 participants, the number the people whose housing were completely damaged were 32, directly affected people by the tsunami were 24, yet these people’s rate of PTSD were almost same as total participants. Therefore, we must look further to understand the mental health impact on those we surveyed.

According to the official report by the Japanese government [28], 80% of the residents within 10km of the power plant did not know the reason why they were required to evacuate, even though the government declared a nuclear emergency. Most of them thought the evacuation would last only several days, and therefore, they only took personal belongings and necessities with them [28]. The evacuees later learned, from television news, the real reason for their evacuation which was the explosion of the nuclear reactors [28].

In the free writing section of our survey, many narratives reported that the repeated television showing of the nuclear disaster led to the development of intense fear, horror or helplessness among the evacuees. Some temporary shelters closed their doors, windows and shut down the curtains, so evacuees were restricted from what was going outside, therefore increasing their sense of isolation. Also, the experience in the shelters was complicated by freezing temperatures, not enough heating, not enough water, food, clothing or beddings. Many victims stayed about one week in the temporary shelter before moving to Saitama prefecture. Hori et al. [29] reported that distress levels were high in the survivors due to a number of factors, including the forced evacuation from their homes and multiple moves among temporary shelters. In a study examining the distress in survivors of the GEJE disaster, they found that 406 deaths in Minamisona City, in Fukushima Prefecture, were officially attributed to disaster related distress [29].

Regarding the impact of the nuclear disaster and radiation contamination, parallels can be drawn to the Chernobyl nuclear disaster. Weisaeth [30] studied the Chernobyl disaster and reported that the explosion in the nuclear power plant contained stressful stimuli typical of shock trauma, and the fear was aggravated by the lack of adequate information immediately after the accident. The residents around Chernobyl were struck with fear and the threat of long-term illness and distrust in political authorities [30]. In a study of depression, suicidal ideation and thyroid tumors among Ukrainian adolescents exposed as children to the Chernobyl disaster by Contis and Foley [31], 115,191 adolescents were screened for depression, suicide ideation, and psychological problems. Using the Children’s Depression Inventory, depression was diagnosed in 15,399 adolescents (13.2%), suicide ideation in 813 (5.3%), and attempted suicide in 354 (2.3%) [31]. Underlying components of the participants' depression were negative mood, interpersonal difficulties, and negative self-esteem [31]. Overall, the group experienced increased prevalence of thyroid cancer, thyroid tumors, depression, and suicide ideation and socioeconomic problems from their relocation from radiation-affected areas [31]. Dougall and Baum [32] report on Three Mile Island nuclear disaster (USA, 1979) in which 150,000 residents were evacuated. The radioactive gas venting directly into the environment 16 months after the accident causing daily threat of contamination and causing many area residents to exhibit elevated symptoms of stress [32].

Reflecting on the high level PTSD symptoms among the Fukushima evacuees, one must consider a number of possible factors. One plausible explanation is the nature of the triple disaster; earthquake, tsunami and nuclear. Because there were no significant differences of PTSD rates between those affected and not by damages to their housing or damage by tsunami in our results (Table 1), the high rate of PTSD symptoms might not be the effect from earthquake and tsunami. It is known that the prevalence of PTSD of natural disasters is often lower than the rates of human-made or technological disasters, and that the prevalence of PTSD following technological disasters ranged from 15% to 75% [2]. Additionally, Bromet [33] examined the emotional consequences of nuclear power plant disasters and found that emotional consequences occur independently of the actual exposure received. He reported that preliminary data from Fukushima suggest that workers and mothers of young children are at risk of depression, anxiety, psychosomatic symptoms and post-traumatic stress disorder as a direct result of their fears about radiation exposure and the indirect result of societal stigma [33].

It is reported that the worst rates of PTSD were from the Piper Alpha oil rig disaster in 1988. Ten years after exposure, 73% of the survivors still showed symptoms of PTSD, and complex psycho-social interrelationships were described in the study [34]. The sinking of a car-ferry in the Baltic Sea in 1994, the MS Estonia, is another tragic human-made disaster. Arnberg F. et al. [35] performed a prospective longitudinal study and found prolonged PTSD remained 14 years after the disaster with the mean IES-R score was 33 and probable PTSD was 27% (Table 3). In this disaster, they suggested the prolonged uncertainty regarding the salvation of the deceased after the event might partially account for the prolonged PTSD found. These cases suggest that the prevalence of PTSD following a by man-made disaster is higher.

Finally, we must consider the social factors that were included in the written narrative section of our survey. Most importantly were the feelings of intense fear, horror and helplessness that were reported by those surveyed.

### Health Status and Social Factors related to PTSD symptoms

With multiple logistic regression analysis, our study indicates the statistically clear influence of the social factors, as well as pre-incident chronic physical and mental diseases on the impact of disaster related stress and subsequent risk of PTSD symptoms.

PTSD has been associated with increased risk of developing hypertension, hyperlipidemia, obesity, coronary heart disease, altered blood coagulation, after adjusting for confounding factors. Other systems as well has been reported as been compromised by PTSD: immune functioning, gastrointestinal conditions, reproductive disorders, musculoskeletal and pain disorders. Higher risks of developing major depressive disorder, panic disorder and substance abuse disorders are also well known [36,37]. The burden added to the pre-incident chronic and mental diseases by PTSD in this sample is predictive of further deterioration of the health status of this affected population if PTSD rates keep high and go to the chronicity too.

Social factors, in particular, were linked to the psychological distress and suffering (Table 2). First was concerns about yet unsolved issues of compensation and reparation (OR = 3.74), the second was worries about livelihood sustainability (OR = 2.27), and the third was loss of jobs (OR = 2.27). These three factors relate to socio-economic stability. Moreover, subsequent delays of the monetary compensation for the nuclear accident make the economic future uncertain, keeping stress and social factors related unsolved. Unemployment’s main cause was displacement and transmigration, and also uncertainty of their future relocation. As the evidence of relocation stress was described by Gerrity et al. [38], our data shows stressful repeated relocation with the average number of relocation was 4.0±1.7 times within one year following the disaster. Evacuees typically relocated to two temporally shelters, one relative’s house as a lodger, and the residence provided temporally by local-government.

It is estimated that most of the evacuees will not be able to return to their homes for about 20 years or more. In other words, they have lost their homes. ‘Home’ is the basis for ‘place attachment’, and it represented an extension of the self, identity with family, and a symbol of the future [38]. In this sense, the evacuees from Fukushima can be considered as serious ‘domestic refugees’ [39] or ‘internally displaced persons, IDPs” [40]. Since this is the first time that IDPs are described in Japan, it is necessary to compare with any conflicts or wars in other country.

The fourth factor is a shrinking of human networks and social ties and stigma associated with being evacuated. The evacuation events destroyed the sustaining bonds between individuals and community. Kukihara et al. [15] speculated the reason for high rates of post-traumatic stress reaction in the Fukushima evacuees was a reaction to displacement, including separation from their families, neighbors and communities. Many narratives recorded in the free description section of the questionnaire included in our survey revealed what amounted to harassment, discrimination and stigma suffered by Fukushima’s evacuated residents, leading them to hide their real origin once relocated in their new neighborhood.

The category of “absence of useful information” for daily refugee life is not strongly enough associated to become a predictor on multiple logistic analysis, even significant difference is shown in chi-square analysis (Table 1). But, it is suggested that the kind of mass information is not adjusted to any particular situation, consequently insufficient to smoothly perform a new life, and it may also include the distrust and unreliable in official information result from ‘information crisis’ [41] after the disaster.

Weisaeth [41] emphasized the several dimensions of serious worries of the victims after Chernobyl as following; sociocultural effects of the displacement, social disruption of communities, psychological aspects of the perception of risk from radiation, role of policies on release of information, socioeconomic dimensions including the return to nonnuclear sources of energy, and finally pathogenic factor relating physiological stress reaction and changes in lifestyle. These are probably related to the greater unpredictability, uncontrollability and culpability in technological disasters [41].

As this study demonstrates the high prevalence of PTSD is strongly related with several socio-economic factors, if these socio-economic factors are not addressed, the chronicity of PTSD symptoms in affected population will likely be prolonged. Therefore, recovery efforts must address not only psychological and physical care but also socio-economic care to achieve recovery from PTSD.

### Limitations of this study

First, our survey was cross-sectional and the cooperation rate was not as high as we would have liked, but our response rate 24.4% is considered to be almost average rate in post disaster studies. All the response rates of the published post disaster studies which used IES-R that we could find using the SCOPUS search engine are shown in Table 3. Although, the studies of public settings were over 50% [7, 10, 11, 15, 21, 22], most of the private setting studies ranged 14% to 33% [8, 9, 26] and three of them used convenience samples [17, 20, 23, 24]. The survivors of huge disasters were most likely too exhausted to complete the survey. Therefore, it is valuable that our data was collected by all the evacuated households temporarily living Saitama prefecture. Second, although there were no statistical difference between the ages older or younger than 60, the mean age of sample was high; 54.2 years of age. Therefore, geriatric sample bias was likely to affect the outcome trend. Third, the mental health responses of this disaster might have been slightly higher due to the one year anniversary reactions [42]. Fourth, since IES-R is a self-response questionnaire to determine the risk of PTSD symptoms, the results do not indicate the diagnosis of PTSD, rather, the risk for PTSD.

## Conclusion

In conclusion, more than factors associated to the earthquake itself, it appears the social factors related to the nuclear disaster and its consequent radiation and evacuation are responsible for the high rate and prevalence of stress in evacuated former residents of Fukushima. Our findings suggest the complex natural (earthquake and tsunami) and technological (nuclear) disaster are significant in the high prevalence of probable PTSD in those surveyed.

The evacuees’ psychological and social suffering simultaneously involve health, welfare, legal, political, economic, and moral issues. Therefore, we suggest that the GEJE, which included the complex natural disasters of the earthquake and tsunami and the subsequent Fukushima disaster with its nuclear radiation leak which triggered the displacement of a large population of people; greatly impacted the survivors. The mental health problems reported by the victims, such as, PTSD, depression and anxiety are best understood in the context of consequences of the disaster; thus fitting the description of post-traumatic stress disorder.

Our findings suggest that any recovery plan for the survivors of the GEJE should address not only health and mental health problems related to the disaster, but should include social issues which were identified in our study, such as, problems related to displacement and job security issues. Therefore, a comprehensive recovery action plan [43] must include policy and programs that address health, mental health and social factors in order to achieve sustainable recovery from complex disasters.

## Supporting Information

S1 Dataset (XLS)
